# Oxalate nephropathy precipitated by linaclotide in a high-risk patient 

**DOI:** 10.5414/CNCS111722

**Published:** 2025-11-05

**Authors:** Omar Ali, Mauricio Monrroy, Andrea Lightle, Giovanni Faddoul

**Affiliations:** 1 Department of Medicine,; 2 Division of Nephrology and Hypertension Care, and; 3 Department of Pathology and Laboratory Medicine, Albany Medical Center, Albany, NY, USA

**Keywords:** linaclotide, oxalate nephropathy, acute kidney injury, drug-induced nephropathy

## Abstract

Introduction: Oxalate nephropathy (ON) is a rare condition caused by calcium oxalate crystal deposition in renal tubules, leading to acute kidney injury (AKI), chronic kidney disease (CKD), or both. The etiologies of ON are divided into two main categories: primary and secondary. Linaclotide is used for the constipation subtype of irritable bowel syndrome (IBS-C), which is not reported to precipitate ON. Case presentation: We report a unique case of linaclotide-precipitated ON in a 50-year-old female with predisposing comorbidities. The patient developed severe AKI (creatinine 8.37 mg/dL) 3 months after starting linaclotide for IBS-C. Symptoms included fatigue, flank pain, and pale stools. Kidney biopsy confirmed ON. Linaclotide was discontinued, and supportive treatment led to significant renal recovery, with creatinine returning to baseline (0.92 mg/dL) within 2 months. Discussion: This case highlights linaclotide’s potential to precipitate ON in patients with risk factors such as malabsorption and dehydration. Secretory diarrhea caused by linaclotide may increase intestinal oxalate absorption, triggering hyperoxaluria. While not inherently nephrotoxic, linaclotide may exacerbate existing susceptibilities. Conclusion: Linaclotide can contribute to ON in predisposed patients. Clinicians should consider medication history and risk factors in unexplained AKI and pursuing kidney biopsy for diagnosis.

## Introduction 

Oxalate nephropathy (ON) is an acute and/or chronic condition in which calcium oxalate (CaOx) crystals accumulate in the renal tubules, disrupting the nephron’s function and impairing renal function. Although infrequent, recent studies have shown that it should not be overlooked as a cause of acute kidney injury (AKI) or chronic kidney disease (CKD), with a prevalence ranging from 1 to 4.07% [1]. 

## Case presentation 

A 50-year-old female presented with an incidental creatinine level of 8.37 mg/dL from a baseline average of 0.9 mg/dL. Her medical history included Sjögren’s disease (limited to arthralgia and dry eye disease), monoclonal gammopathy of undetermined significance (MGUS), IgG κ paraproteinemia, diabetes mellitus type 2 (DM type 2), Roux-en-Y gastric bypass surgery (RYGB surgery), and irritable bowel syndrome with constipation (IBS-C). She reported a 1-week history of increased fatigue, bilateral flank pain, urinary frequency, low-grade fevers, decreased appetite, and nausea. Her elevated creatinine was noted during her bi-weekly plasmapheresis for small-fiber polyneuropathy. She reported increased urine output without dysuria or discoloration and denied any recent illnesses, nephrolithiasis, or any personal or familial history of kidney disease. She reported occasional use of NSAIDs for pain, approximately once a week. She denied alcohol use and did not smoke except for the occasional use of marijuana. The only recent change in her medications was the initiation of linaclotide 72 µg oral capsule twice daily 3 months ago for IBS-C. The patient endorsed changes in her stools 1 – 2 weeks after starting linaclotide. In addition to increased frequency of bowel movements (the desired effect), her stools had changed in character, becoming pale and pasty, which was unusual. Physical examination revealed bilateral costovertebral tenderness and obesity with a BMI of 35 kg/m^2^. 

## Investigations 

Initial labs indicated hyperchloremic metabolic acidosis, mild leukocytosis (9.6 × 10^3^/µL), elevated erythrocyte sedimentation rate (ESR) of 46 mm/h, C-reactive protein (CRP) of 89.5 mg/L, alkaline phosphatase (ALP) of 119 U/L, and fibrinogen levels of 819 mg/dL. Urinalysis showed increased eosinophils without any crystals. 

Urine studies demonstrated an osmolality of 216 mOsm/kg, protein of 13 mg/dL, urea nitrogen of 204 mg/dL, sodium of 63 mEq/L, and creatinine of 46.1 mg/dL. Simultaneous serum measurements showed potassium of 4.1 mEq/L, sodium of 140 mEq/L, and creatinine of 8.37 mg/dL. Severe AKI can occur in lieu of non-oliguric urine output [2]. Precise urine output was not documented during the first 2 days of admission, mainly due to staffing limitations. However, the patient continued to produce urine during her stay, with output ranging from 0.9 to 3 L per day. A 24-hour urine collection was deemed inappropriate in this setting, given the presence of AKI. Typically, a 24-hour urine collection is obtained on an outpatient basis to assess conditions such as urinary stone disease, proteinuria, and multiple myeloma [3]. Additionally, AKI disrupts the steady state of solute concentration in both serum and urine, which would have resulted in an erroneous estimation of solute concentration in the urine [4]; also, ON was not high on the list of differential diagnoses at the time of admission. 

A renal ultrasound was negative for hydronephrosis or kidney stones. The urine drug screen was positive for benzodiazepines and cannabinoids, both of which the patient confirmed using actively. Further serology showed negative antinuclear antibodies (ANA), negative hepatitis C serology, negative anticardiolipin, β-2 glycoprotein, IgG, IgM, and IgA, and normal λ light chains and ratio. Further review of an 8-year period of previous health records within another healthcare system showed one urinalysis 2 years prior to this presentation showing calcium oxalate crystals. Also, the patient’s urine pH has been consistently between 5 and 6.5 except for one occasion when it was 7 during the 8 years preceding this presentation, which covers the entirety of the period after RYGB surgery. Similar review of serum electrolytes during the same period demonstrated normokalaemia and normal bicarbonate levels. 

## Treatment, outcome, and follow-up 

Upon admission, the patient was managed supportively with intravenous hydration by lactated Ringer’s solution, monitoring of intake and output, and sodium bicarbonate supplementation to address the acidosis. Additionally, the nephrology team was consulted. Given the patient’s history of Sjögren’s disease, glomerulonephritis (GN) was high on the differential diagnosis list, and serological studies were ordered. The patient’s urine was examined manually under microscopy, revealing muddy brown casts with non-dysmorphic red blood cells. The presence of eosinophils in the urine raised concerns for acute interstitial nephritis, possibly due to one of her medications. The decision to obtain a kidney biopsy was discussed with the patient, and she consented to the procedure, which was performed on hospital day 5. 

**Figure 1.**
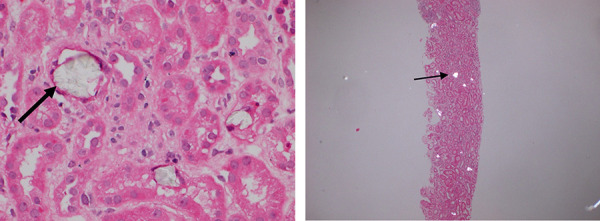
Left: Tubular injury associated with luminal fan-like translucent crystals (× 400); Right: Polarization highlights numerous birefringent luminal crystal (× 40).

Linaclotide was discontinued on hospital day 6 (before the biopsy results were available) after further history revealed she had developed pale and pasty stools after starting the medication. Her renal biopsy revealed numerous birefringent CaOx crystals within the proximal tubular lumina, in association with patchy sloughing of tubular epithelial cells, apical blebbing, interstitial edema, and mononuclear and eosinophilic inflammation, with minimal chronic changes (< 5% fibrosis/atrophy), characteristic of acute ON (Figure 1). There have been instances where scarce oxalate crystals were found in kidney biopsies from patients without ON; this has led Buysschaert et al. [5] to suggest adding an oxalate crystals-to-glomeruli ratio ≥ 0.25 in biopsy to the definition for ON. This patient met all the criteria for the definition of ON, including AKI, widespread deposition of CaOx crystals in the tubular epithelial cells and tubular lumina, exclusion of other causes of AKI, and the presence of a hyperoxaluria-enabling condition [1, 5, 6]. 

**Figure 2.**
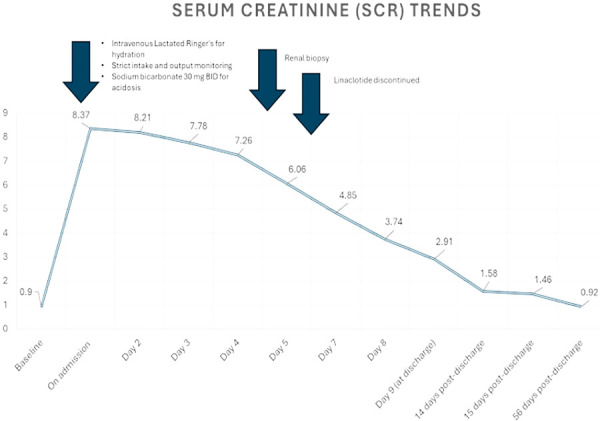
Creatinine baseline is around 0.8 mg/dL. The drawing shows the creatinine trends starting 2 weeks prior to patient’s presentation till day 70.

On discharge, the patient was advised to stay hydrated and was prescribed calcium tablets to chelate oxalate and advised to adhere to a low-oxalate diet. At a follow-up visit 1 week after discharge, her creatinine had improved to 1.46 mg/dL. After 2 months, her creatinine had normalized to 0.92 mg/dL (Figure 2). 

## Discussion 

We present the case of a 50-year-old female with a complex medical history, including comorbidities commonly associated with ON. The recently added linaclotide precipitated an AKI due to acute ON, which was confirmed by biopsy. A thorough literature review revealed no previously reported correlations between linaclotide and ON. This case demonstrates the delicate balance between ingestion and enteric excretion of oxalate and the development of hyperoxaluria leading to ON. It also highlights an unexpected side effect of linaclotide, shedding light on the potential of new medications to cause enteric hyperoxaluria. Further caution is warranted when prescribing new medications to populations with underlying comorbidities that could lead to ON. Thus, it is essential to remain vigilant and investigate medications in any unexplained onset of renal injury in patients without previous renal disease or those with acutely progressing CKD after years of stability. 

ON mainly occurs due to hyperoxaluria. The etiologies for hyperoxaluria (Table 1) can be divided into primary (overproduction) and secondary causes (increased ingestion, absorption, and decreased elimination). 

Primary hyperoxaluria (PH) is a group of autosomal recessive inherited inborn errors of metabolism of oxalate’s precursor (glyoxylate) in the liver. There are three genotypes of PH depending on which enzyme is deficient in the glyoxylate pathway. PH type 1 is the most common and severe genotype, accounting for 80% of PH cases. PH onset peaks during childhood with a median onset age of 5.5 years. PH type 1 occurs due to a deficiency of hepatic alanine glyoxylate aminotransferase (AGT), which functions to convert glyoxylate to glycine. PH type 2 and PH type 3 occur due to deficiencies in the enzyme glyoxylate reductase/hydroxypyruvate reductase (GR/HPR) and mitochondrial enzyme 4-hydroxy 2-oxoglutarate aldolase, respectively. Additionally, oxalate deposition in these conditions is systematic and not limited to the kidneys. 

Secondary causes include increased ingestion of dietary oxalate or its precursors (glycine, serine, tyrosine, tryptophan, ethylene glycol, and ascorbic acid), fat malabsorption or steatorrhea (secondary to any cause leading to fat malabsorption such as short bowel syndrome, RYGB surgery, chronic pancreatitis, pancreatectomy, Crohn’s disease, medication-related), and decreased breakdown of ingested oxalate due to reduced intestinal colonization with *Oxalobacter formigenes* and other species. Additionally, etiologies that cause decreased estimated glomerular filtration rate (eGFR), such as volume loss, ACE inhibitors and diuretics, and aging, have all been linked to ON [1, 5, 6, 7]. 

Enteric hyperoxaluria refers to enteric pathology leading to fat malabsorption and hyperoxaluria. Only 5 – 10% of dietary oxalates are absorbed in the bowels due to the majority being bound by dietary calcium. Nevertheless, increased fat content causes decreased available calcium to bind to free oxalate, leading to increased absorption and thus increased hyperoxaluria. In recent years, this has been more prevalent due to the development of multiple bariatric surgeries that contribute to fat malabsorption, such as jejunoileal bypass and RYGB surgery. Additionally, increased bile salts and fatty acids increase the local membrane permeability, thus facilitating further absorption of oxalate. One increasingly important aspect of enteric hyperoxaluria is the degradation of oxalate by bacteria, including *Oxalobacter, Bifidobacterium,* and *Lactobacillus* species. The use of antibiotics may disrupt the gut microbiome and decrease the number of oxalate-degrading species, further contributing to the incidence of ON. Furthermore, medications used to treat hyperlipidemia, such as orlistat, and medications that may impact gastrointestinal motility, permeability, or fat absorption can all play a role in disrupted oxalate metabolism. Some literature has shown an association between DM, obesity, and increased oxalate excretion in urine [8, 9]. 

**Figure 3.**
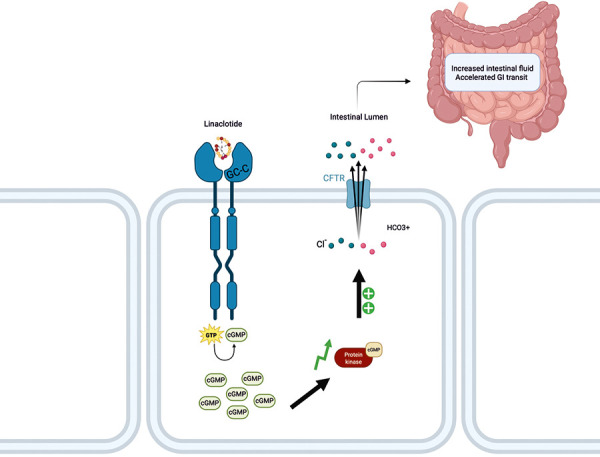
Linaclotide’s mechanism of action [19].

Linaclotide is a drug approved for the treatment of IBS-C and chronic constipation (Figure 3). It is a 14-amino-acid synthetic peptide that works by activating the guanylyl cyclase-C (GC-C) receptor located on the intestinal epithelial cells, leading to increased fluid secretion in the intestinal lumen along with increased gastrointestinal transit [10]. It is similar to the endogenous peptide hormones guanylin and uroguanylin. Diarrhea is the most common adverse effect and is often reported within the first 4 weeks of starting linaclotide [10, 11]. 

Chronic diarrheal illness has been associated with ON, and there exist two case reports describing ON associated with *Clostridium difficile* *(C. diff)*.It is postulated that *C. diff* may lead to ON due to increased permeability of the intestinal mucosa, leading to increased oxalate absorption, fat malabsorption in the setting of small bowel involvement, and alteration of intestinal flora of oxalate-metabolizing bacteria [12, 13]. 

The patient’s known comorbidities of RYGB surgery, recurrent volume depletion secondary to plasmapheresis, DM type 2, and obesity are all known factors and comorbidities that are associated with ON. Additionally, her historic urine analysis showed CaOx crystals, which demonstrates an already established state of subclinical hyperoxaluria, waiting for any trigger to tilt the scales into a manifest state of disease. While crystalluria is an important clue that may further guide management, it is common to have an acute presentation of ON without the presence of crystalluria [14, 15]. A case series of 12 patients with biopsy-proven ON without crystalluria on presentation illustrates this diagnostic challenge [16]. Another case series of 11 patients with ON included only 3 patients presenting with crystalluria [17]. According to the literature, patients with a predisposition to hyperoxaluria, such as those who have undergone RYGB, may develop ON at highly variable time intervals, with a mean time to diagnosis of 8 years. Additionally, approximately two-thirds of such patients were found to present with clinical steatorrhea at the time of diagnosis [5]. In this case, we believe that the recent development of steatorrhea shortly after the initiation of linaclotide may have precipitated this patient’s episode of ON. This conclusion is supported by the mechanism of action of linaclotide, which induces secretory diarrhea by activating the GC-C receptor. We theorize that the increase in secretory fluids and decreased transit time leads to a reduced contact time between oxalate and calcium in the gastrointestinal tract, increasing the availability of free oxalates for absorption. Additionally, the chronic diarrheal effect of linaclotide can alter the microbiome and decrease the number of oxalate-degrading bacteria, which in turn increases the number of luminal free oxalates. This cascade can independently cause hyperoxaluria. A similarity can be noted between *C. diff* and linaclotide’s side effects that lie in the incidence of (watery/secretory) diarrhea despite different mechanisms. This further corroborates and links diarrheal etiologies and ON. The association between ON and linaclotide can essentially be attributed to fat malabsorption and increased oxalate absorption. 

While it is possible that the patient’s initial metabolic acidosis could be attributed to distal renal tubular acidosis (dRTA) associated with Sjögren’s syndrome, given that the prevalence of dRTA in patients with Sjögren’s is estimated to range between 5 and 25% [18], this diagnosis typically presents with recurrent calcium phosphate nephrolithiasis and/or chronic metabolic acidosis with persistently alkaline urinary pH. Our patient’s historical laboratory data did not demonstrate chronic metabolic acidosis or sustained alkaline urine pH, and has been normokalemic historically. In addition, the patient had no known history of nephrolithiasis. Calcium phosphate crystals typically appear as non-birefringent basophilic material and are often associated with chronic nephrocalcinosis; our biopsy showed strongly birefringent fan-shaped CaOx crystals within acutely damaged tubules, supporting ON rather than phosphate-related injury [1]. Furthermore, the patient’s clinical history is more suggestive of a predisposition toward hyperoxaluria rather than hypercalciuria, corroborating a diagnosis of secondary ON rather than dRTA-related nephrocalcinosis. While we do not believe there is enough evidence in this case to demonstrate that linaclotide can cause ON on its own, we do think that linaclotide can precipitate ON when used in the presence of other risk factors. We also note that although the patient’s creatinine began to normalize while she was still taking linaclotide, we emphasize that crystal deposit nephropathies are not usually a continuous state, but rather an intermittent event triggered by a perfect combination of dehydration, appropriate pH, and component saturation leading to crystal deposits. Crystallopathies should not be approached in the same way as interstitial nephritis secondary to medication toxicity. 

## Conclusion 

Our case highlights that extensive history taking remains of the utmost importance when working up acute renal failure of unknown etiology and without any prior kidney disease. As novel therapies continue to emerge, we are bound to see novel adverse effects of these therapies. A renal biopsy is often necessary in these situations to confirm the etiology. Linaclotide’s adverse effect of diarrhea is well established. However, its downstream effects can lead to unforeseen complications in already predisposed patients. 

## Data availability 

Access to data is permitted with the authors’ permission. 

## Authors’ contributions 

M. M. contributed to the concept and study design. A. L. contributed to data acquisition, images, and analysis. O. A. contributed to data interpretation and drafting of the manuscript. O. A. and G. F. provided critical revisions. G. F. supervised the project. M. M., A. L., O. A., and G. F. take responsibility that this case report has been presented honestly, accurately, and transparently, and accept accountability for the overall work by ensuring that questions pertaining to the accuracy or integrity of any portion of the work are appropriately investigated and resolved.

## Funding 

The authors declare that no funds, grants, or other support were received during the preparation of this manuscript.

## Conflict of interest 

The authors declare that they have no conflict of interest. 

**Table 1.** Causes of oxalate nephropathy.

Category	Cause	Description
Primary causes	PH type 1	Deficiency of hepatic alanine glyoxylate aminotransferase (AGT)
	PH type 2	Deficiency of glyoxylate reductase/hydroxypyruvate reductase (GR/HPR)
	PH type 3	Deficiency of mitochondrial enzyme 4-hydroxy-2-oxoglutarate aldolase
Secondary causes	Increased oxalate ingestion	High dietary intake of oxalate-rich foods
	Fat malabsorption	Conditions like short bowel syndrome, RYGB surgery, chronic pancreatitis
	Reduced intestinal colonization	Decreased oxalate-degrading bacteria (e.g., *Oxalobacter formigenes*)
	Reduced eGFR	Conditions causing decreased filtration rate (e.g., volume loss, ACE inhibitors, diuretics, aging)

PH = primary hyperoxaluria; eGFR = estimated glomerular filtration rate; RYGB = Roux-en-Y gastric bypass.
